# Critical Review of the Evolution of Extracellular Vesicles’ Knowledge: From 1946 to Today

**DOI:** 10.3390/ijms22126417

**Published:** 2021-06-15

**Authors:** Erica Bazzan, Mariaenrica Tinè, Alvise Casara, Davide Biondini, Umberto Semenzato, Elisabetta Cocconcelli, Elisabetta Balestro, Marco Damin, Claudia Maria Radu, Graziella Turato, Simonetta Baraldo, Paolo Simioni, Paolo Spagnolo, Marina Saetta, Manuel G. Cosio

**Affiliations:** 1 Department of Cardiac, Thoracic, Vascular Sciences and Public Health, University of Padova, 35128 Padova, Italy; mariaenrica.tine@gmail.com (M.T.); alvise.casara@gmail.com (A.C.); davide.biondini@unipd.it (D.B.); umberto.semenzato@gmail.com (U.S.); ecocconcelli@icloud.com (E.C.); elisabetta_balestro@hotmail.com (E.B.); marco.damin@gmail.com (M.D.); graziella.turato@unipd.it (G.T.); simonetta.baraldo@unipd.it (S.B.); paolo.spagnolo@unipd.it (P.S.); marina.saetta@unipd.it (M.S.); manuel.cosio@mcgill.ca (M.G.C.); 2 Department of Women’s and Children’s Health, University of Padova, 35128 Padova, Italy; claudiamaria.radu@unipd.it; 3 Department of Medicine, University of Padova, 35128 Padova, Italy; paolo.simioni@unipd.it; 4 Meakins-Christie Laboratories, Respiratory Division, McGill University, Montreal, QC H3A 0G4, Canada

**Keywords:** exosomes, microvesicles, multivesicular bodies, liposomes

## Abstract

Extracellular vesicles (EVs) are a family of particles/vesicles present in blood and body fluids, composed of phospholipid bilayers that carry a variety of molecules that can mediate cell communication, modulating crucial cell processes such as homeostasis, induction/dampening of inflammation, and promotion of repair. Their existence, initially suspected in 1946 and confirmed in 1967, spurred a sharp increase in the number of scientific publications. Paradoxically, the increasing interest for EV content and function progressively reduced the relevance for a precise nomenclature in classifying EVs, therefore leading to a confusing scientific production. The aim of this review was to analyze the evolution of the progress in the knowledge and definition of EVs over the years, with an overview of the methodologies used for the identification of the vesicles, their cell of origin, and the detection of their cargo. The MISEV 2018 guidelines for the proper recognition nomenclature and ways to study EVs are summarized. The review finishes with a “more questions than answers” chapter, in which some of the problems we still face to fully understand the EV function and potential as a diagnostic and therapeutic tool are analyzed.

## 1. Introduction

Extracellular vesicles (EVs) are a family of particles released from the cell that are delimited by a lipid bilayer and cannot replicate, i.e., do not contain a functional nucleus, and can be detected in culture supernatants of different bodily fluids [1,2,3,4,5]. Once considered nothing else than sticky cell debris, they are now recognized as alternative mediators of cell communication, being able to convey a broad range of molecules such as cytokines, inflammatory mediators, and miRNA [1,2,3,4,5,6], crucial in the maintenance of homeostasis, induction/dampening of inflammation, and promotion of repair [1,2,3,4,5,6]. Recognized today as “extracellular vesicles” (EVs), they were initially stratified and nominated based on their size from <100 nm, traditionally named exosomes; 100 to 1000 nm, also recognized as microvesicles, microparticles or ectosomes; and apoptotic bodies when >1000 nm [7], a nomenclature that was eventually modified after the MISEV 18 guidelines.

**Figure 1:**
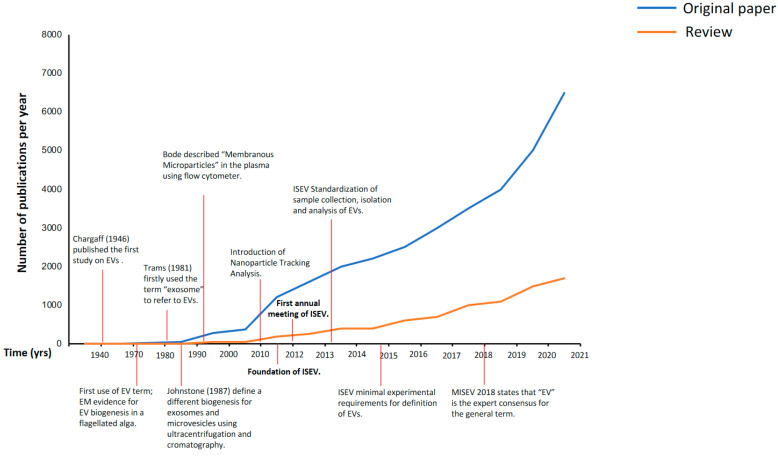
Number of publications per year in PubMed from 1946 to 2021. After the first annual meeting of the International Society of Extracellular Vesicles (ISEV) in 2012, the number of original papers and reviews including any extracellular-vesicle-related terms (exosomes, microvesicles, microparticles, and extracellular vesicles) increased exponentially. EVs: extracellular vesicles; EM: electron microscopy; ISEV: International Society of Extracellular Vesicles; MISEV: Minimal Information for Studies of Extracellular Vesicles.

As shown by the number of publications (Figure 1), there has been an exponential increase in the interest in EVs and their potential applications in understanding the underlying mechanisms of various diseases, such as cancer, cardiovascular, metabolic, neurological, and infectious diseases, among others, which have revealed a role for EVs as promising biomarker candidates for diagnosis, prognosis, and even therapeutic tools, in lung and other diseases [8,9,10,11]. Furthermore, investigations performed with cell cultures of peripheral and epithelial cells from various systems highlight the peculiar release of specific cargoes of these particles when exposed to different triggers, which points to the possibility that EVs might be bioengineered as shuttles of therapeutic drugs, offering an alternative way of drug delivery for disease treatment [9,11].

Currently, the most striking evidence of their importance comes from the study of circulating human EVs in inflammatory disorders, and even more from the recognition of their presence in body fluids [12]. EV-containing “liquid biopsies” such as blood [13], BAL [14], urine [15], saliva [13], and cerebrospinal fluid [16] can be obtained in an easy and minimally invasive way and are seen as a promising alternative to regular biopsies [9]. Indeed, the comprehensive characterization of EVs and of their content in body fluids might offer the key to better understand disease development and treatment.

## 2. Evolution of the EVs Nomenclature: From 1946 to Today

As shown in Figure 1, recent decades have seen a sharp increase in the number of scientific publications describing the physiological and pathological functions of “extracellular vesicles” (EVs), a collective term covering various subtypes of cell-released membranous structures referred to as exosomes, microvesicles, microparticles, ectosomes, oncosomes, apoptotic bodies, and many other names. Unfortunately, these terms, even today, mean different things to different investigators, which creates vexing problems related to their nomenclature. Furthermore, specific issues arise when working with these entities, whose size and amount often make them difficult to obtain as relatively pure preparations and to characterize properly.

A brief description of the development of the knowledge and nomenclature of EVs might help to understand the evolution of the field, possibly clarify the nomenclature, and expose the issues that need to be reviewed in order to progress in the understanding and clinical use of EVs [1,17].

The existence and possible function of EVs were initially suspected in 1946 by Chargaff and West while studying thromboplastin and platelets [18]. They hypothesized that, in addition to the thromboplastic agent, a “variety of minute breakdown products of the blood corpuscles” contributed to the clotting properties [18]. In 1967, Wolf [19] investigated and provided evidence showing that the responsible plasma coagulant material, hypothesized by Chargaff as “minute breakdown products of the blood corpuscles”, was indeed minute particulate material rich in lipid content originating from platelets, as clearly shown by electron microscopy (EM) and referred to as “platelet-dust”. It should be thus considered that it was Wolf who provided the first description of not only EVs but also of their function in the specific conditions investigated.

The term “intracellular and extracellular vesicles” was first used in 1971 by Aaronson et al. [20], who, by using EM, gave the first ultrastructural evidence for the production of a wide variety of intra- and extracellular membranous structures in *O. danica*, a flagellated alga. Importantly, Aaronson showed that vesicles and other membranous structures arose from several cell organelles, which clearly recognized EV biogenesis as a biological process and not a fixation artefact [20]. They also described how intact vesicles could be recovered by centrifugation. However, despite these first observations, throughout the 1970s, the lack of precise information on the biology of EVs raised important questions about the origin of these membranes, specifically whether they come from viral shedding out of the cell or from random debris of dead cells during ultracentrifugation or are actually released from living cells in a more or less specific process.

Studies in the late 1970s using EM and chemical lysis (nitrogen cavitation) identified in fetal bovine serum numerous elongate and spherical microvesicles with a trilaminar membranous envelope ranging from 30 to 60 nm. These structures were shown to be either normal cell components or the breakdown products of normal cell components, not to be confused with viruses. They were classified as “plasma membranes”, “extracellular membranes”, and “microvesicles” according to the compartment of origin [21].

Later studies further confirmed that they were not virus-like particles nor cell debris but represented a biological entity derived from intra- and extracellular compartments; however, no further information on the biological origin, content, and function of EVs was given [22].

The term exosome (“exo” = outside and “soma” = body) was already introduced in the 1970s [23,24,25], referring to DNA fragments transferred between cells. However, the lack of association of the DNA fragments with lipid bilayers probably prevents these particles from being considered an early description of exosomes or extracellular vesicles. Not until 1981 was the term “exosome” proposed to refer to the exfoliation of “microvesicles” from the plasma membrane by Trams et al. [26]. These authors have contributed important advances to the knowledge and understanding of EV genesis and function. In particular, the fact that exfoliation of membranous vesicles might occur in many different normal and neoplastic cells and that the exfoliative process is selective strongly suggested, based on their lipid composition, that microvesicles consist of specific domains of the plasma membrane [26]. Interestingly, with the use of EM, they described two populations of vesicles, one of which consisted of irregularly shaped vesicles approximately 500 to 1000 nm in diameter which contained another population of smaller, spherical vesicles with an average size of about 40 nm. This was very likely the first description of what later was recognized as a multivesicular body (MVB), the source of exosomes. They also found that constituents of the microvesicles could be transferred to recipient cells and suggested the possibility that the shedding of microvesicles and their interaction with a target cell or target organ represented a physiological phenomenon that takes place in vivo. Conceivably, the vesicle could be incorporated into the recipient cell, thereby producing a modification of the host cell. Of interest, Trams proposed, in 1981, the visionary suggestion of using the liposomes, which contain the “exosome” cargo, to target therapeutic use, a vision that has become a reality for the COVID-19 vaccines [27].

Although Trams’ publication was often cited during the 1980s, the term “exosome” was not used again to refer to EVs until 1986 [28] and again in 1987 by Rose Johnstone and colleagues [29] after they had described in reticulocytes the formation of multivesicular structures, which, after their fusion with membranes, released the vesicles they contained into the medium. By this mechanism, the reticulocyte could rid itself of proteins and structures that were no longer required in order to become a mature red cell [30]. These authors were the first to report about exosomes as intraluminal vesicles of multivesicular endosomes that were secreted upon fusion with the plasma membrane [31], defining for the first time different biogenesis pathways for exosomes and microvesicles [32].

Initially described as a means to extrude obsolete components by a very specific cell type, the reticulocyte, exosomes remained minimally investigated for the following 10 years. In addition, most cell biologists remained skeptical about the actual existence of this “weird” secretion pathway and were convinced that exosomes were merely membrane fragments artificially released upon in vitro cell handling. Subsequent to their discovery in reticulocytes, the MVB structures and exosomes were eventually shown to be present in many cell types, including B lymphocytes in 1996 [33], dendritic cells in 1998 [34], platelet [35] and epithelial cells in 1991 [36], and neurons in 2005 [37]. Owing to the original findings that the MVB/exosome complex could have an important role in the immune activation and stimulation of adaptive immune responses [33,34], a renewed interest in exosomes arose in the immunology field, which provided essential information about their generation and biology [38]. It is now recognized that the generation of exosomes, with budding, fission, and segregation occurring within the MVB lumen, does not occur by default but is governed by specific processes. Furthermore, the discharge of exosomes has been shown to take place not only by constitutive but also by regulated exocytosis of MVBs activated in response to specific intracellular signals [39]. Nowadays, exosomes are described in mammals and invertebrates and appear to be involved in many different processes [40,41,42,43].

### 2.1. Exosomes or Microvesicles or Extracellular Vesicles (EVs)

**Figure 2:**
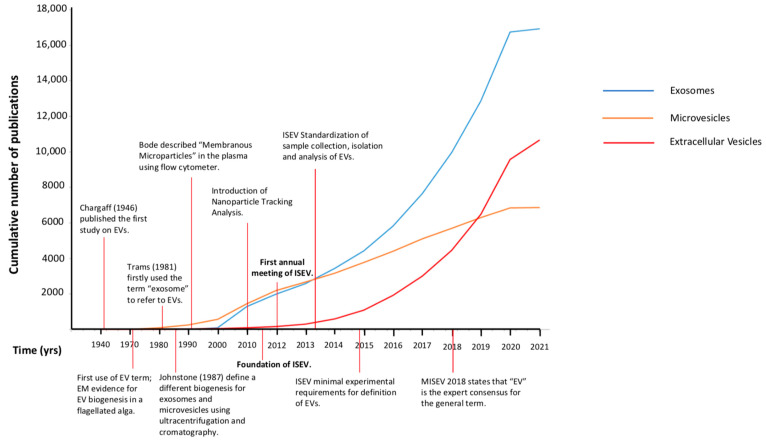
Cumulative number of publications. Comparative evolution of the use of different terms for EVs (exosomes, microvesicles or extracellular vesicles) in the literature from 1946 to 2021. EVs: extracellular vesicles; EM: electron microscopy; ISEV: International Society of Extracellular Vesicles; MISEV: minimal information for studies of extracellular vesicles.

The technological outgrowth started in the 1990s, which introduced new methods facilitating the study of EVs beyond the EM (nanoparticle-tracking analysis, dynamic light scattering, high-resolution flow cytometry), contributing to a progressive expansion of the scientific knowledge and publications on the field (Figure 1 and Figure 2). However, researchers continued to refer to EVs indiscriminately as either exosomes or microvesicles but also as ectosomes, membrane particles, exosome-like vesicles, and apoptotic vesicles [38]. In the period from the late 1990s to the early 2010s, the growth of the curves of the scientific production referred to as “exosomes” or “microvesicles” were completely overlapped (Figure 2), as much as the two terms were in researchers’ minds.

It was Gyorgy et al. [7] in 2011 who, aiming to clarify the confusion created by the heterogeneity of vesicle terminology, suggested that the large number of mobile membrane-limited vesicles contained in the extracellular environment should be termed “extracellular vesicles” and not microparticles, as particle suggest a solid, particulate structure rather than a vesicular one. EVs would include exosomes, activation- or apoptosis-induced microvesicles/microparticles, and apoptotic bodies [7]. They also proposed, to make the terminology unambiguous, to classify EVs into three broad classes based on their biogenesis, secretory components, and size: exosomes, microvesicles, and apoptotic bodies. Exosomes are 50–100 nm EVs of endosomal origin containing certain surface markers including tetraspanins. Microvesicles are larger than exosomes (100–1000 nm) and are derived from the plasma membrane of cells through direct outward budding. They contain membrane components, as do their parent cells. Apoptotic bodies are released from cells that undergo apoptosis and can be 50–5000 nm in diameter. They may contain DNA fragments, noncoding RNAs, and cell organelles [7].

By 2013, Gould and Raposo [44] highlighted how the expanding interest in EVs had also introduced some vexing problems related to their nomenclature and how the proposed generic terms meant different things to different investigators. The term exosome has been used, and still is nowadays, in three different ways: some investigators following the original biogenetic definition (i.e., vesicles that bud into endosomes and are released when the resulting MVBs fuse with the plasma membrane [29,33]); others preferring the original, broad definition (i.e., secreted vesicles that ‘‘may serve a physiologic function’’ [26,45]); and others still employing an empirical definition based on differential centrifugation (i.e., vesicles that sediment only after centrifugation at 70,000–100,000 *g* [29,46]). A similar range of definitions was evident for the term microvesicle, which some define as vesicles that bud from the plasma membrane [38,47], others define as all secreted vesicles [48], and others still define on the basis of differential centrifugation (i.e., vesicles that sediment at 10,000 *g* [38,46]).

It is of interest how the term exosome gained popularity among investigators, as evidenced by the increasing number of publications over the years referring to exosomes (Figure 2). Probably the fact that the exosome, along with the MVB, were the EVs most studied and more clearly described attracted its use, even when it might not have been appropriated. The description of MVBs in immune cells and how the resulting exosomes had a function in adaptive immunity gave the exosomes a relevance never reached by “microvesicles” being mere pieces of the cell membrane [49].

Soon after the description of MVBs and their 40 to 80 nm exosomes cargo by Johnstone in 1985 [30], differential centrifugation and ultracentrifugation above 100,000 *g* was used in order to isolate and differentiate the smallest vesicles, the exosomes, from EVs larger than 100 nm, the microvesicles, and apoptotic bodies [31]. This technique was used broadly, alone or along with EM and other techniques, to study the properties, biological content, and identification markers of these smallest EVs, the exosomes. Extensive literature has revealed how exosomes can be identified by the expression of endosome tetraspanins (CD37, CD53, CD63, CD81, and CD82, among others), which are possibly responsible for cell penetration, invasion and fusion events [50,51]. Furthermore, in 2007 Valadi et al. [52] showed that exosomes obtained from human mast cell line cultures, identified by ultracentrifugation and expression of CD63 by FACS analysis, contained RNA from approximately 1000 genes, many of which were not present in the cytoplasm of the donor cell. Further analysis revealed also the presence of miRNA, which was transferable to other human and mouse cells. However, in addition to exosomes, various other membrane-derived vesicles (~50–4000 nm in diameter) in the circulation were also found to contain miRNA [53].

### 2.2. The International Society for Extracellular Vesicles (ISEV) 2013: First Attempt to Introduce Some Order into EV Science

As the interest and consequent number of publications on EVs, mainly exosomes (the ‘‘buzz term’’ for EV-related science), increased, by 2013, it was becoming evident that most studies were not clearly defining the origin of EVs under study, neither were they defining the relative contributions of exosomes and of other secreted membrane vesicles in the proposed functions. It was by then clearly recognized that a major ongoing challenge was to establish methods that would allow discrimination between exosomes and microvesicles, as differences in properties such as size, morphology, buoyant density, and protein composition seemed insufficient for a clear distinction. Paradoxically, the increasing interest for the content and function had progressively reduced the relevance for a precise nomenclature in classifying EVs, therefore leading once again to a confusing scientific production. In response to this confusion, in 2013, the International Society for Extracellular Vesicles (ISEV), a group of scientists with collective long-term expertise in the field of EV biology, proposed a series of criteria, based on best current practice, that represented the minimal characterization of EVs that should be reported by investigators. The idea was that adoption of these criteria should aid researchers in planning studies and reporting their results. In addition, the ISEV’s recommendations suggested appropriate controls that should be included in EV-related functional studies [2,3].

In order to systematically review the impact of the 2013 ISEV criteria for the characterization of EVs in scientific production, the EV-TRACK knowledgebase consortium was convened to record experimental parameters of EV-related studies [5]. A review of a checklist of 115 parameters based on the MISEV guidelines, related to sample type, preanalytical variables, isolation protocol, and characterization method in 1742 experiments published in 2010–2015, revealed widespread heterogeneity in EV isolation methods and inconsistent reporting of important experimental parameters [1,2,3,5]. Moreover, it is important to underline that only 18% of experiments include both qualitative and quantitative analysis, and 50% did not achieve more than 20% of EV–METRIC (a checklist to assess the completeness of reporting of generic and method-specific information necessary to interpret and reproduce the experiment, according to Reference [5]). These analyses revealed that a large number of publications on EVs contained insufficient information for unambiguous interpretation or replication of experiments [5].

### 2.3. The Minimal Information for Studies of Extracellular Vesicles (MISEV) 2018

It was amply apparent and worrisome to experts within the ISEV community that the continuous large increase in EV publications often reported major conclusions that were not sufficiently supported by the experiments performed or the information reported. These circumstances prompted a revision and renewal of the MISEV recommendations that brought to date the new knowledge in the area, along with a commitment by the ISEV to continue to work toward their wider acceptance and implementation. We think it is important at this point of the review to summarize the salient conceptual recommendations of the MISEV 2018 [1].

#### 2.3.1. Nomenclature

ISEV endorses “extracellular vesicles” as the generic term for particles naturally released from the cell that are delimited by a lipid bilayer. Not a consensus on specific markers of EV subtypes, such as endosome-origin “exosomes” and plasma-membrane-derived “ectosomes” (microparticles/microvesicles), authors are urged to consider the use of operational terms for EV subtypes that refer to:

(a) Physical characteristics of EVs, such as size (“small EVs” and “medium/large EVs”, with ranges defined, for instance, respectively, as <100 nm or <200 nm (small), or >200 nm (large and/or medium)) or density (low, middle, and high, with each range defined);

(b) Biochemical composition (CD63+/CD81+ EVs, Annexin A5-stained EVs, etc.);

(c) Descriptions of conditions or cell of origin in the place of terms such as exosome and microvesicle that are historically burdened by manifold/contradictory definitions and inaccurate expectations of unique biogenesis.

#### 2.3.2. On the Characterization of EVs by their Protein Composition

The described rich sources of potential EV subtype specific markers are acknowledged in the guidelines. However, because experiments were performed with different approaches and using different cellular sources, it is not possible to propose specific and universal markers of the different types of EVs, let alone of MVB-derived “exosomes” as compared with other small EVs. Consequently, MISEV 2018 does not propose molecular markers that could characterize specifically each EV subtype.

#### 2.3.3. Specific versus Common Functions of Different Types of EVs

An important point to keep in mind is that, when analyzing exclusively the function of a single type of EV, for instance either small EVs or large EVs (that are called ectosomes, microvesicles, or microparticles in different studies), one may miss the most active EV subtype for the particular function studied. Even if a function is found in the concentrated small EV preparation, it could also be present, and even possibly more concentrated, in other EV subtypes that had been eliminated during the small EV isolation process. Keeping large EVs (e.g., “microvesicles”) and comparing their activity to that of small EVs should be a first step in all functional studies [1].

#### 2.3.4. Determine whether a Function Is Specific to Exosomes, as Compared with other Small EVs

It is now clear that different types of EVs can present functional activities that are as important to explore as those elicited by late endosome-derived exosomes. However, in the last decade, many studies have focused exclusively on demonstrating the association of a given function with exosomes. The technical limitations of such studies, and why they are not sufficient to conclude, as is generally done, that exosomes have specific functions compared with other EVs, are highlighted in the guidelines [1].

## 3. More Questions than Answers

The MISEV18 guidelines recommended that the term EVs should be used to refer to both small EVs, exosomes, and medium/large EVs, microvesicles, as differences in properties such as size, morphology, buoyant density, and protein composition seem insufficient for a clear distinction as an operational term. However, considering their origin, “exosomes” ought to be different than the EVs “microvesicles”, and a major and important challenge for the future would be to establish methods that will allow the discrimination between MVB/exosomes and microvesicles. Exosomes were initially viewed simply as a means by which cells discarded unwanted proteins, lipids, and “junk RNA” [54]. Recent data have challenged this view, and it is now believed that cells use exosomes as “messengers” or “biological drug carriers” to exert specific effects on their environment [55,56].

**Figure 3:**
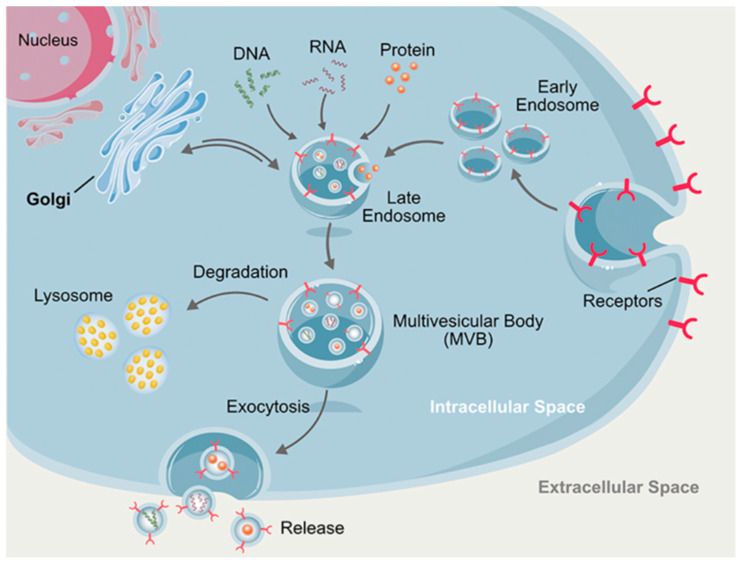
Biogenesis and cargoes of exosomes. Exosome development begins with endocytosis to form early endosomes, later forming late endosomes by inward budding, and finally generating multivesicular bodies (MVBs). MVBs could then either undergo degradation (generating lysosomes) or merge with the cell membrane and, by exocytosis, release intraluminal endosomal vesicles that become exosomes into the extracellular environment. Reprinted with permission from Reference [51].

Exosomes are different. It is well accepted that the “real” exosomes [51] are small EVs, which are formed by a process of inward budding in early endosomes, later forming multivesicular endosomes and eventually multivesicular bodies (MVBs) (Figure 3), which after fusion with the cell membrane release exosomes into the extracellular microenvironment to transfer their components [39,57].

**Figure 4:**
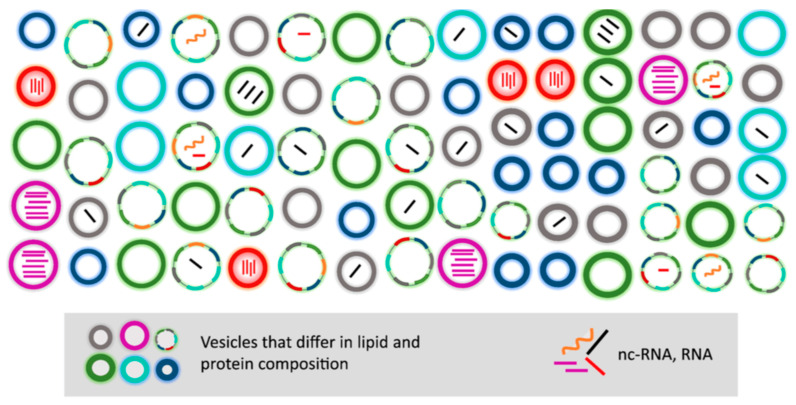
Proposed model of heterogeneous exosomal composition and subpopulations. Different subspecies exist within exosomes isolated from the same batch of cells. Exosomes can differ with regard to lipid, protein, and miRNA composition. “Potent” exosomal subspecies enriched with a high number of miRNA (depicted as purple and red vesicles) exist next to exosomes that are essentially devoid of nucleic acids. Reprinted with permission from Reference [54].

In most cases, exosomes are produced and released “on demand”, following activation of a cell surface receptor. Exosome composition is not a mere copy of cytosolic content; rather, it can change in response to different stimuli [58,59,60]. Such changes in exosome composition determine the final outcome of exosome-mediated communication [61,62]. Exosomes derived from the same cells have traditionally been expected to contain a similar protein, nucleic acid, and lipid composition. However, it has recently been shown that the molecular composition of exosomes is not only cell-type dependent but can differ even when the exosomes originate from the same parental cells (Figure 4) [63,64,65]. It is becoming apparent that both the subcellular origin of exosomes and donor cell activation status can contribute to their molecular heterogeneity [63,64,65].

Part of exosome “molecular heterogeneity” might be contributed by the lysosomal exocytosis. Lysosomes are terminal degradative organelles whose functions are fundamental to reduce intracellular stress and preserve cellular homeostasis through clearance of damaged or toxic material, including proteins, lipids, and even nucleic acids [66]. Conceivably this cargo could add further confusion to the sorting of the definition of the active molecules contained in the exosomes (Figure 4).

The heterogeneity of exosomes has to be critically analyzed with regard to the limitations of isolation techniques [54]. This is because most current methods to collect exosomes result in bulk isolates. Current analytical methods, including Western blotting and proteomic methods, are suitable for bulk isolate characterization but cannot distinguish the properties of individual vesicles; differences in the molecular composition of specific subpopulations are masked during isolation and processing and are not readily detectable [54].

To that end, novel methods have very recently been developed that might capture exosomal heterogeneity [60,67,68]. In particular, high-resolution flow cytometry (also known as micro flow cytometry), which relies on the optimization of commercially available flow cytometers, has been used for this purpose. With this technique, several authors, [60,68,69] using a dual labeling approach for the analysis of exosomal heterogeneity in different experiments, were able to detect significant differences in either MHC-II and MFG-E8 [70] or tetraspanin CD63 and EpCAM [68] expression in exosomes. These results strongly suggest that bulk vesicle extracts contain different exosome subspecies either randomly or in spatially distinct patterns. Using laser tweezers Raman spectroscopy, Smith and colleagues demonstrated that exosomes derived from the same cells consist of several populations of exosomes that differ in protein and lipid membrane composition [64]. As well as protein heterogeneity, other species including nucleic acids, especially miRNAs and mRNAs, are packaged in exosomes and are likely to show similar variability (Figure 4). These findings also strongly indicate that the biological effects mediated by exosomes are the sum of all components, i.e., the correct combination of lipids, proteins, and nucleic acids. Understanding the role of each component would be essential to understand how their combination contributes to the overall biological outcome. Because exosomes are mostly analyzed as bulk isolates, their heterogeneity in composition and population is often overlooked. It still remains a challenge to isolate specific populations of exosomes, and technological advances are urgently required to address this problem.

Most of the recent basic research on EVs is focused on MVB-derived exosomes, with little or no attempts in investigating the membrane-derived 100 to 1000 nm EVs (“microvesicles”). It seems at present there is no possible way to clearly differentiate “microvesicles” from “exosomes” by either morphology, size, or function; thus, it is recommended that all EVs should be considered as a single entity. However, the fact that we cannot differentiate them once they are released from the cell does not necessarily mean that they are all the same. They ought to be different, but we cannot tell them apart. Exosomes are clearly “fabricated” on demand to serve a specific function by some specific cells, mainly immune cells, that can form MVB/endosomes later released as exosomes with different biological content according to various stimuli. To complicate matters further, it is known that exosome cargo might vary, not only with the cell producing the exosomes but also by the different endosomes/exosomes produced by the same cell.

It is said that most cells produce MVBs, but it is difficult to find literature evidence of other cells than immune and reticulocytes in which MVBs and the derived exosomes are clearly demonstrated. Much of the new research performed on the definition of exosomes has been conducted using cell cultures of mainly immune cells (T and B cells, mast cells, and dendritic cells) and malignant cells using ultracentrifugation of supernatants, and it could be questioned if the separation of MVB-derived exosomes and membrane-derived vesicles can be differentiated by these procedures in cell cultures. However, the use of cell cultures could help to clarify and differentiate the source of EVs exosomes versus microvesicles. The exosome composition and content seem to be controlled by posttranslational modifications such as ubiquitination. Interferon-stimulated gene 15 (ISG15) is an interferon (IFN)-induced ubiquitin-like protein that covalently conjugates proteins. Protein modification by ISG15 (ISGylation) is known to inhibit the replication of many viruses, and, interestingly enough, it has been found that ISGylation also inhibits exosome production and secretion [58]. Conceivably, preventing the production of exosomes in cell culture after cell stimulation with IFN would induce ISGylation and suppression of exosomes. The resulting EVs would then be “microvesicles”, the properties of which could then be investigated.

It is difficult to imagine that vesicles derived from an “established” structure, such as a cell membrane, could have the same potential for biological variation as the endosome/MVB/exosome complex has. It would be easier to imagine that MVB-derived exosomes and membrane-derived EVs could work “in tandem” and cooperate in facilitating the essential signal delivered by the exosome to reach the intended target.

## 4. Conclusions

This review of the literature on EVs has shown the important evolution in the knowledge in the area and highlighted the extreme importance and complexity of cellular and intercellular communication by the use of EVs in the maintenance of homeostasis and response to challenges. Finding consensus in the field is complicated by methodological challenges such as the use of different methods for exosome isolation and quantification. However, this is an important and interesting field that needs to be further explored. Moreover, considering the role of exosomes in physiological and pathological conditions, strategies that interfere with the release of exosomes and impair exosome-mediated cell-to-cell communication could potentially be exploited therapeutically in the future. The use of exosomes to create the mRNA-based COVID-19 vaccine, an idea already suggested by Trams in 1981, points the way to the importance of further deciphering the role of EVs and exosomes in health and disease.

## Data Availability

Not applicable.
